# Local *Salmonella* Enteritidis restaurant outbreak investigation in England provides further evidence for eggs as source in widespread international cluster, March to April 2023

**DOI:** 10.2807/1560-7917.ES.2023.28.27.2300309

**Published:** 2023-07-06

**Authors:** Helen E Benson, Lucy Reeve, Lucy Findlater, Amoolya Vusirikala, Maaike Pietzsch, Oluwakemi Olufon, Eve Matthews, Ann Hoban, Anaïs Painset, Incident Management Team, Sooria Balasegaram, Lesley Larkin, Sarah Weir, Ellen Heinsbroek, Incident Management Team, Richard Brown, Krystal Carpenter, Julia Manuel, Agnieszka Bird, Tina Potter, Clare Maguire, Janice Lo, Amy Douglas

**Affiliations:** 1 East of England Health Protection Team, UK Health Security Agency, Cambridge, United Kingdom; 2 Field Service East of England, UK Health Security Agency, Cambridge United Kingdom; 3 Field Service South East and London, UK Health Security Agency, London, United Kingdom; 4 Field Service Rapid Investigation Team, UK Health Security Agency, London, United Kingdom; 5 Gastrointestinal Infections and Food Safety (One Health) Division, UK Health Security Agency, London, United Kingdom; 6 Gastrointestinal Bacteria Reference Unit, Public Health Microbiology Division, UK Health Security Agency, London, United Kingdom; 7 Watford Borough Council, Watford, United Kingdom; Food Standards Agency, London, United Kingdom; and UK Health Security Agency, London, United Kingdom

**Keywords:** *Salmonella* Enteritidis, Whole Genome Sequencing, Outbreaks, Restaurants, Epidemiology, food-borne

## Abstract

We report a 5-single nucleotide polymorphism cluster of *Salmonella* Enteriditis in England, part of a global cluster of *S.* Enteritidis ST11. Forty-seven confirmed cases have been investigated of whom 25 were linked to a restaurant. In addition, there were 18 probable cases with restaurant exposure. Epidemiological investigations suggested eggs or chicken as the most likely cause of the outbreak but were unable to distinguish between those two food vehicles. Ongoing food chain investigations indicated links to imported eggs from Poland.

An outbreak of food-borne infection linked to a restaurant was reported to the UK Health Security Agency (UKHSA) East of England Health Protection Team (HPT) in early April 2023. Whole genome sequencing (WGS) results indicated *Salmonella* Enteritidis infection, with all cases in a 5-single nucleotide polymorphism (SNP) cluster falling into a wider genomically diverse 10-SNP cluster investigated in several countries. We identified additional cases in the 5-SNP cluster with no known links to the restaurant, and historical cases reported since July 2022. 

Our investigations aimed to define common exposures for cases in the 5-SNP cluster to determine the likely source of infection and implement control measures.

## Case definition

A confirmed case was defined as a person with laboratory-confirmed *S.* Enteritidis infection belonging to the 5-SNP cluster 1.2.3.18.180.7268.% [1] in England since 1 March 2023. A probable case was defined as a person with gastroenteritis or confirmed *Salmonella* spp. infection in England who dined at the restaurant of interest from 1 March to 1 April 2023. Confirmed cases were further categorised as having known, unknown or no exposure to the restaurant.

## Outbreak description

In early April 2023, UKHSA was notified via Accident and Emergency doctors, general practitioners, and a local authority environmental health (EH) department of multiple cases of gastroenteritis following food consumption at a restaurant, with attendance or take-away dates in late March. We identified *S.* Enteritidis as the causative organism. *Salmonella* isolates are routinely sent to the UKHSA Gastrointestinal Bacteria reference unit (GBRU) for sequencing [2]. The outbreak cases were confirmed to be within a 5-SNP cluster defined at UKHSA as 1.2.3.18.180.7268.% matching the Enterobase cgMLST hierarchical cluster HC2_316378 [3], falling into a wider genomically diverse cluster 1.2.3.18.180.%/HC5_2301 that is subject to several national and international investigations.

Cases identified through initial case notifications were interviewed using generic food history questionnaires; for cases notified since 2 May 2023, a bespoke menu-based questionnaire was used. Early cases were re-interviewed with the bespoke questionnaire completed by telephone or online. Additional cases identified through WGS were contacted to establish whether they were linked to the restaurant, and if so, asked to complete the bespoke questionnaire by telephone or online. Cases who did not reveal exposure to the restaurant completed a modified *Salmonella* trawling questionnaire focussed on poultry products.

## Descriptive epidemiology

**Figure 1:**
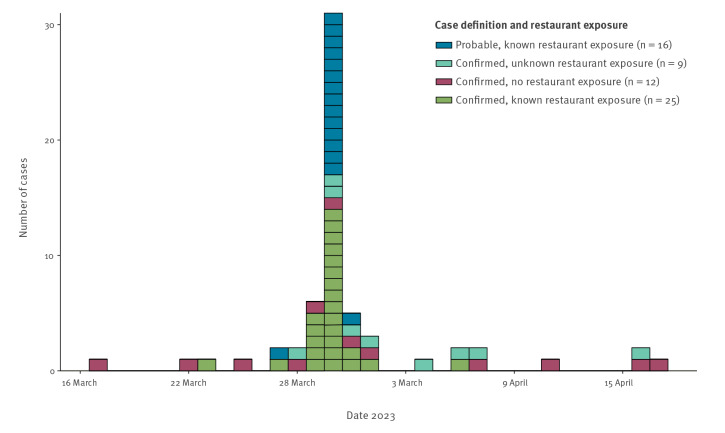
Cases of *Salmonella* Enteritidis by case definition and onset date or presumptive onset date, England, March–April 2023 (n = 62)*

There were 65 cases associated with this 2023 outbreak; 25 confirmed and 18 probable cases linked to the restaurant, 10 confirmed cases with unknown link to the restaurant and 12 confirmed cases with no link to restaurant (Figure 1). 

Onset date used if available (n = 37), otherwise presumptive onset date used (n = 25). Date was not available for one confirmed and two probable cases; these are not included in the figure.

In total 20 bespoke menu-based questionnaires were completed (17 by phone, three online), 12 generic food history questionnaires were completed (seven online, five by phone), six modified *Salmonella* trawling questionnaires were completed (three online, three by phone), and there were 27 cases for whom a notification was made but no questionnaire was completed (15 were contacted but did not complete a questionnaire and 12 were not contactable after the initial notification).

Onset dates ranged from 17 March to 17 April 2023; this includes presumptive onset dates for cases with only a specimen date available (calculated as the specimen date minus the median number of days between specimen date and onset date for cases with complete information (5 days, range: 3–18 days)). Most cases experienced onset of symptoms on 30 March 2023.

Symptoms included vomiting, diarrhoea, fever and abdominal pain, with onset 12–24 h following food consumption from the restaurant. Ten cases were hospitalised (10/34 with information available) with a total of 49 seeking healthcare (49/51 with information available).

**Figure 2:**
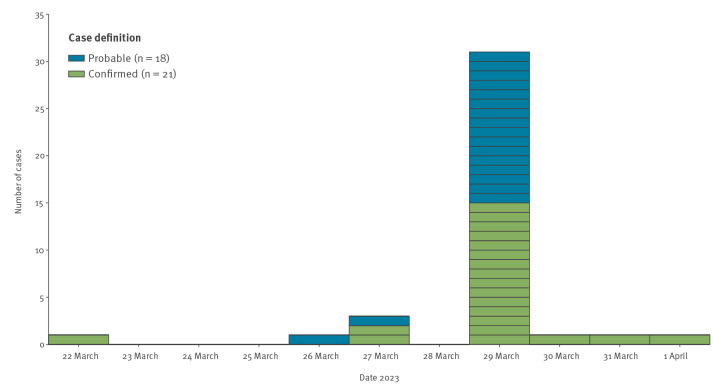
Cases of *Salmonella* Enteritidis linked to a restaurant, by case definition and dining date, England, March–April 2023 (n = 39)

For the 43 restaurant-linked cases, the mean age was 32 years (range: 6–61 years) and 17 were female (sex collected as binary variable; unknown for two cases). The majority of cases were resident within the same region as the restaurant. Dining dates ranged from 22 March to 1 April 2023, with the majority dining on 29 March 2023 (Figure 2). There were no cases reported among restaurant staff members.

Dining date was unavailable for four of 43 restaurant−linked cases.

Resident postcodes for confirmed cases with no link to the restaurant or where this was not known were more widely geographically distributed.

## Analytical studies

To enable a case–control study, we tried to recruit controls via a ‘snowballing’ method, by asking cases to nominate members of their dining parties who did not experience any gastrointestinal symptoms. This method failed to recruit a sufficient number of controls as almost all cases reported that all their fellow diners experienced symptoms.

Instead, we used restaurant till receipt data to calculate attack rates and odds ratios for specific menu items. Till receipt data have been used successfully in previous outbreak investigations [4,5]. The till receipt data available for this restaurant only included the total number sold of each dish, no individual food orders. We restricted the analysis to purchase data from 29 March. We assumed that each individual ordered each dish only once to provide an estimate of the number of total diners exposed. From the number of main dishes sold, we estimate that a total of 451 diners on 29 March were included in the analysis. Further analysis used grouped menu items to calculate attack rates and odds ratios for exposure to common ingredients. We restricted the grouped analysis to main dishes to avoid duplicate counting of the total number of diners. For the exposed cases, we restricted the analysis to the 31 cases who had eaten food from the restaurant on 29 March. Food exposure data from the menu-based questionnaire was available for 19 cases; limited food exposure data from the generic food questionnaire or original notification was available for 12 cases.

Odds ratios were elevated for any dish containing egg, any dish containing chicken and any dish possibly containing egg (comprising any dish containing egg and any dish served with a choice of sauce including mayonnaise or garlic mayonnaise containing egg) (Table). Eating chicken or possibly eating egg would explain, respectively, 25 or 24 of the 31 cases. We could not identify whether chicken or egg was the more likely source of infection as all cases who definitely ate egg also ate chicken, and all cases who ate chicken who reported an exact dish consumed a dish served with naan or a sauce containing egg. Multivariate analysis was not possible without control questionnaire data.

**Table:** Results of univariable analysis on exposure to restaurant food items using till receipt data, England, March–April 2023 (n = 451^a^)

Exposure	Number of cases exposed	Number of total diners exposed	Attack rate (%)	Odds of case among exposed	OR	95% CI	p value^b^
*Any dish containing chicken*	25	270	9.26	0.10	2.92	1.17–7.27	0.014
*Any dish possibly containing egg*	24	261	9.20	0.10	2.61	1.10–6.19	0.024
*Any dish served with mayonnaise or garlic sauce*	21	225	9.33	0.10	2.16	0.99–4.70	0.042
*Any dish definitely containing egg*	19	130	14.62	0.17	4.38	2.06–9.31	< 0.001
Non-vegetarian snacks	15	198	7.58	0.08	1.19	0.57–2.47	0.708
*Any dish containing lamb*	10	219	4.57	0.05	0.50	0.23–1.09	0.065
Sundries	8	281	2.85	0.03	0.19	0.08–0.44	< 0.001
Chicken tikka roll	7	10	70.00	2.33	40.48	9.85–166.42	< 0.001
Flame chicken meals	5	81	6.17	0.07	0.93	0.35–2.50	1.000
Full flamed chicken with chips	3	16	18.75	0.23	3.34	0.90–12.41	0.089
BBQ and tandoori dishes	2	41	4.88	0.05	0.66	0.15–2.87	1.000
Rice dishes	2	74	2.70	0.03	0.36	0.08–1.54	0.139
Vegetable snacks	2	38	5.26	0.06	0.79	0.18–3.45	1.000
Chicken doner roll	2	12	16.67	0.20	2.83	0.59–13.52	0.196
Half chicken with chips	2	45	4.44	0.05	0.65	0.15–2.82	0.757
2 Kebabs with chips	1	27	3.70	0.04	0.53	0.07–4.04	1.000
Chicken kebab roll	1	4	25.00	0.33	4.59	0.46–45.48	0.249
Mixed kebab roll	1	3	33.33	0.50	6.97	0.61–79.09	0.193
Chicken tikka with chips	1	16	6.25	0.07	0.95	0.12–7.44	1.000
Quarter chicken with chips	1	17	5.88	0.06	0.81	0.10–6.32	1.000
Four chicken kebabs	1	5	20.00	0.25	3.47	0.38–32.03	0.301
Half chicken	1	2	50.00	1.00	13.97	0.85–228.93	0.133

CI: confidence interval; OR: odds ratio.

^a^ Total number of diners exposed estimated on number of main dishes in till receipt data.

^b^ p values calculated using Fisher’s exact test.

Grouped variables items shown in italics. The Table shows only individual dishes with at least one case exposed.

## Food and case exposure investigations

When EH officers visited the restaurant on 4 April 2023, there were no remaining food items to sample. They identified no lapses in food safety or hygiene concerns such as cross-contamination issues or inadequate cooking of chicken. The EH officers identified that the restaurant used raw eggs to make garlic mayonnaise and as a binder ingredient in naan bread.

Food exposure information was provided to the Food Standards Agency, who undertook food chain investigations. This identified that the eggs used at the restaurant were purchased from wholesalers who had imported the eggs from Poland. Food chain investigations for two cases with no link to the restaurant also identified that they consumed eggs imported from Poland. One case purchased and ate eggs from a local independent store selling imported food; the food tracing investigation identified that these eggs had been imported from Poland. The second case ate a meal containing eggs at a different restaurant; food chain analysis identified that the eggs used in this restaurant were also imported from Poland. Information on the source of the chicken is pending.

## Control measures

The narrow window of dining dates for restaurant-linked cases suggested a contaminated batch of food or an isolated lapse in procedures. General food safety advice was reiterated to the food business operator, including a recommendation to change the supply of eggs to domestically sourced eggs produced under a recognised farm assurance scheme.

The investigation team continues to monitor WGS results; as of 30 June, there have been two additional cases in this cluster.

Information on the evidence suggesting eggs imported from Poland to be the likely source of this outbreak has been communicated to the relevant Polish health authorities.

## Discussion

*Salmonella* spp. are a major cause of food-borne outbreaks, and poultry products are regularly implicated in *S*. Enteritidis outbreaks [6]. There is a notable public health value in rapid multi-country sharing of epidemiological, food tracing and microbiological data, and the increasing use of WGS allows not only improved strain discrimination but also improved detection of cross border threats and additional analysis options informed by the epidemiological and food supply chain contexts [6-8].

This outbreak investigation is of high relevance due to the international context. Since 2014, Europe has seen an internationally distributed cluster defined at UKHSA as 10-SNP cluster 1.12.13.18.180.% matching the Enterobase cgMLST hierarchical cluster HC5_2301 [3]. This large cluster has been associated with consumption of chicken meat or eggs from multiple origins across Europe [8,9]. Our outbreak sequences belong to a specific 5-SNP cluster identified as 1.2.3.18.180.7268.%/HC2 _316378 which constitutes a genomic subset of the endemic 10-SNP cluster 1.2.3.18.180.%/HC5_2301. The common exposure of these cases to a single restaurant over a short period of time provided an opportunity to elucidate potentially implicated food chains.

The rise in cases within this 5-SNP cluster indicates that there could be further outbreaks elsewhere. An event was posted on the EpiPulse information exchange platform hosted by the European Centre for Disease Prevention and Control (event ID 2023-FWD-00034). Similar sequences to our representative sequences were posted by Austria and Poland on Enterobase; they also belong to the HC2_316378 cluster, indicating closely genetically related human cases in those countries.

Our analytical investigation was restricted by insufficient recruitment of controls, thus we used till receipt data. To analyse these aggregate data, we assumed that each individual ordered each dish only once to provide an estimate of the number of total diners exposed. It is, however, likely that some individuals ordered multiple items of the same category. In our analysis we could also not account for anecdotal reports of items being shared by diners. Another limitation is that our calculation of attack rates and odds ratios assumes that diners not identified as cases did not have gastrointestinal symptoms. This assumption will have underestimated the attack rates and odds ratios; it is likely that there were more undetected cases given the high number of cases identified among fellow diners of cases when we tried to recruit controls. Given the overlap in menu items, it was not possible to separate consumption of chicken from eggs, and elevated odds ratios were identified for both; it is possible that the elevated odds observed for chicken are due to confounding given the overlap in menu items, but it is also possible that consumption of chicken and eggs were independently associated with illness: either through cross-contamination at the restaurant, or due to potential widespread contamination within multiple chicken sectors. While chicken consumption cannot be ruled out as the source at this stage, the high odds ratio for definite egg consumption in combination with the food chain investigation results would suggest that the eggs were the main source of the restaurant outbreak.

## Conclusion

Epidemiological and ongoing food chain investigations suggest that imported eggs from Poland are the likely source of this outbreak. We anticipate that this investigation into a discreet clade within the wider cluster phylogeny will contribute to the evidence base to better understand and control the sources of this complex international cluster.

## *Erratum

In the version originally published on 6 July, the date 3 April on the x axis of Figure 1 was erroneously written as 3 March. This was corrected on 25 July 2023. We apologise for any inconvenience this may have caused.
