# Erratum for Euro Surveill. 2023;28(27)

**DOI:** 10.2807/1560-7917.ES.2023.28.30.230727e

**Published:** 2023-07-27

**Authors:** Eurosurveillance editorial team

**Affiliations:** 1 European Centre for Disease Prevention and Control (ECDC), Stockholm, Sweden

In Figure 1 of the article entitled ‘Local Salmonella Enteritidis restaurant outbreak investigation in England provides further evidence for eggs as source in widespread international cluster, March to April 2023’ by Benson et al., published on 6 July 2023, the date 3 April on the x axis was erroneously written as 3 March. This was corrected on 25 July 2023. We apologise for any inconvenience this may have caused.

